# Detection of macrovesicular steatosis in hematoxylin and eosin-stained histological images of human livers: A feature-based method

**DOI:** 10.1016/j.jpi.2026.100656

**Published:** 2026-03-27

**Authors:** E. Malamutmann, M. Platte, S. Theurer, M. Jalc, J. Rashidi-Alavijeh, K. Willuweit, J.-W. Treckmann, A. Oezcelik, F. Nensa, J. Haubold, U. Neumann, D.P. Hoyer

**Affiliations:** a General, Visceral & Transplantation Surgery, University Hospital Essen, Hufelandstr. 55, 45147 Essen, Germany; b Clinic for Gastroenterology and Hepatology, University Hospital Essen, Hufelandstr. 55, 45147 Essen, Germany; c Institute of Pathology, University Hospital Essen, Hufelandstr. 55, 45147 Essen, Germany; d Institute of Artificial Intelligence, University Hospital Essen, Hufelandstr. 55, 45147 Essen, Germany; e Institute of Radiology and Neuroradiology, University Hospital Essen, Hufelandstr. 55, 45147 Essen, Germany

**Keywords:** Automated image analysis, Macrovesicular steatosis (MaS), Liver steatosis quantification, Whole-slide imaging (WSI), Digital pathology, Liver histology, Image processing techniques, Liver transplantation, Donor liver evaluation

## Abstract

**Background:**

Macrovesicular steatosis (MaS) affects liver transplant outcomes. Traditional visual biopsy assessment is subjective and shows inter-observer variability (weighted κ = 0.595 in our cohort), which complicates allocation and prognostication.

**Methods:**

We developed a semi-automated image analysis method using HALCON Progress (evaluation license, 2019–2023) to quantify MaS in H&E whole-slide images (WSIs). The pipeline processes native SVS files at full resolution (average runtime 1.26 ± 0.53 min/WSI) without tiling, downsampling, or format conversion. Four shape features (area, roundness, circularity, and compactness) guide classification. Results were compared with three specialized pathologists using Pearson and Spearman correlations.

**Results:**

Across 129 WSIs (≈52,000 lipid droplets), artificial intelligence–pathologist correlations were statistically significant (Pearson *r* = 0.526–0.642; Spearman ρ = 0.498–0.615; all *p* < 0.001; *n* = 48–95 per pathologist). Correlation with the mean pathologist assessment reached R^2^ = 0.64 (*r* = 0.80, ρ = 0.782), within the inter-pathologist range (R^2^ = 0.34–0.62; weighted κ = 0.595). Using four shape features, the pipeline separates vacuoles from vessels and processing artifacts during interactive review. Processing native SVS at full resolution avoids extra compute while preserving precision.

**Conclusions:**

The method provides a rapid, objective readout of MaS and performs on par with inter-pathologist agreement (R^2^ = 0.64 vs. weighted κ = 0.595). It is best used as decision-support that supplies percentages and overlays for expert review. Main limitations are underestimation in severe, confluent steatosis (>30%), limited coverage of microvesicular fat, and single-center validation. Multi-center evidence across scanners and patient groups is still needed before routine use.

## Introduction

Liver steatosis is a key determinant of donor organ quality in transplantation. Lipid accumulates in hepatocytes as macrovesicular (large droplets displacing the nucleus) or microvesicular (small droplets with the nucleus preserved) patterns.^1^ Macrovesicular steatosis (MaS) raises the risk of ischemia–reperfusion injury, so a quantitative readout is required for donor evaluation.^2^ Grafts with moderate (31–60%) or severe (>60%) MaS carry higher risks of early allograft dysfunction and primary non-function, sometimes leading to re-transplantation or recipient death.^1^, ^3^, ^4^, ^5^

Routine assessment—pathologist inspection of hematoxylin and eosin (H&E)-stained frozen sections—faces inconsistent sampling, subjective grading, and time pressure in transplant workflows.^2^, ^3^, ^4^, ^6^, ^7^, ^8^ Macroscopic impressions correlate poorly with histology, and risk tools such as the NAFLD fibrosis score or BAR score only indirectly reflect lipid burden.^9^, ^10^, ^11^ Inter- and intra-observer variability remains substantial and can drive discordant utilization decisions, whereas the scarcity of transplantable grafts pushes centers toward narrow safety margins.^12^, ^13^

Digital pathology enables large-scale image analysis with better consistency and throughput.^14^, ^15^, ^16^, ^17^ Clinical deployment still meets practical barriers: staining variability, validation needs, and fit with routine workflows that have little time for specialist tools.^18^, ^19^, ^20^ Classical morphometric pipelines that rely on color thresholds drift with stain changes and artifacts.^3^, ^21^, ^22^ Machine- and deep-learning approaches can be accurate but often require large annotated datasets, GPU hardware, and opaque models.^23^, ^24^, ^25^, ^26^ Approaches that favor interpretability are a workable middle ground, yet few papers document the full workflow needed for reproducible adoption.

We therefore developed a transparent, feature-based workflow for MaS quantification on H&E whole-slide images (WSI). The system combines morphology-aware preprocessing with interpretable shape descriptors, enabling rapid deployment on CPU workstations while retaining direct pathologist oversight. Crucially, the workflow is intended as a quantitative adjunct that provides objective percentages and visual overlays for pathologists; it is not positioned as an autonomous binary classifier for clinical decision-making. The following sections describe the donor cohort, algorithmic pipeline, and validation strategy that support reproducible adoption in time-critical transplantation settings.

## Material and methods

### Donor data

In the initial stage of the study, researchers conducted a retrospective analysis of the clinical and histological data of organ donors who contributed livers. The primary aim of this study was to evaluate macro- and microvesicular steatosis in donor livers. Data were collected from 1394 organ donors spanning 2010–2019, with histopathological assessments carried out at the Institute of Pathology, University Hospital Essen, and the findings were digitally archived. The Eurotransplant database served as the data source. Anonymization was implemented, and a matching process was performed using the donor number to correlate the examination data with digital records from the Institute of Pathology. Due to incomplete documentation, the dataset was reduced to 1105 cases, accounting for 80% of the original data.

### Clinical donor data

Using the donor number, donor clinical data were retrieved from the Eurotransplant database. In each case, 66 variables were identified. Cases from 2010 to 2019 submitted by the German Organization for Acquisitions (Deutsche Stiftung Organtransplantation, DSO) containing liver tissue were identified from the digital database of the Institute of Pathology. Cases lacking liver tissue or beyond the diagnostic scope of steatosis (e.g., evaluation of cysts, space-occupied lesions, and changes in the bile duct) or without steatosis information were excluded. Cases before 2010 without digital documentation of histopathological examination were also excluded. Among the remaining 1105 cases, a diagnostic pathologist assigned the degree of macro- and microvesicular steatosis.

### Digital whole-slide images and validation cohort selection

#### Slide selection and digitization

From the 1105 donors with complete clinical data, a subset of 129 frozen liver biopsy slides (routinely performed immediately before transplantation) were selected for digitization through the institutional biobank. Selection prioritized slides with available pathologist steatosis assessments performed during routine diagnostic workflow between 2010 and 2019. All slides were scanned at 20× and 40× magnification using an Aperio scanner and saved in SVS format (pyramidal multi-resolution structure, maximum resolution 0.249 μm/pixel). Image dimensions ranged from 3984–63,744 pixels width (mean 40,690 ± 10,720) to 19,311–84,728 pixels height (mean 41,876 ± 12,713), with file sizes of 120–1500 MB (mean 526.6 ± 301.9 MB).

### Halcon image processing software and processing time

HALCON offers multiple APIs (HALCON, C++, C#, C, Visual Basic, and Python); we used the HDevEngine interface so complete HDevelop pipelines or individual procedures could run inside host applications. HDevelop itself acted as our optimization console: multi-gigabyte SVS slides open at full resolution, threshold sliders update overlays instantly, and intermediate steps (deconvolution, thresholding, and morphology) remain visible for debugging. A senior liver transplant pathologist iteratively adjusted area and diameter limits, roundness, circularity, compactness, and related artifact filters while reviewing overlays on representative training slides until the segmentation reflected expert judgement; these training slides were separate from, and not included in, the 129-slide validation set. The resulting standard settings reported in Table S2 were then applied unchanged to all 129 validation slides.

Processing ran on HALCON Progress (evaluation license, 2019–2023) installed on an AMD Ryzen Threadripper 2920× workstation (24 cores @ 3.5 GHz, 64 GB RAM, Ubuntu 24.04 LTS). The XL hrunxl mode loads SVS files natively, avoiding tiling, resampling, or format conversion and the patch-stitching artifacts they introduce. A benchmark across 126 slides yielded a mean runtime of 1.26 ± 0.53 min per WSI (median 1.15 min, range 0.28–2.70 min, 95th percentile 2.3 min) and a strong linear relationship with file size (Time [s] = 23.40 + 0.0997 × FileSize [MB], *r* = 0.928, R^2^ = 0.862, *p* < 0.001), keeping analysis well within the 30-min frozen-section window.

**Fig. 1:**
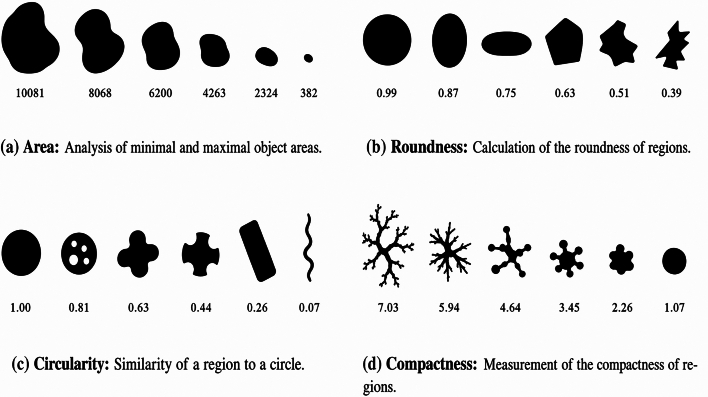
Comparison of object-specific features. (a) Area: Analysis of minimal and maximal object areas. (b) Roundness: Calculation of the roundness of regions. (c) Circularity: Similarity of a region to a circle. (d) Compactness: Measurement of the compactness of regions.

**Fig. 2:**
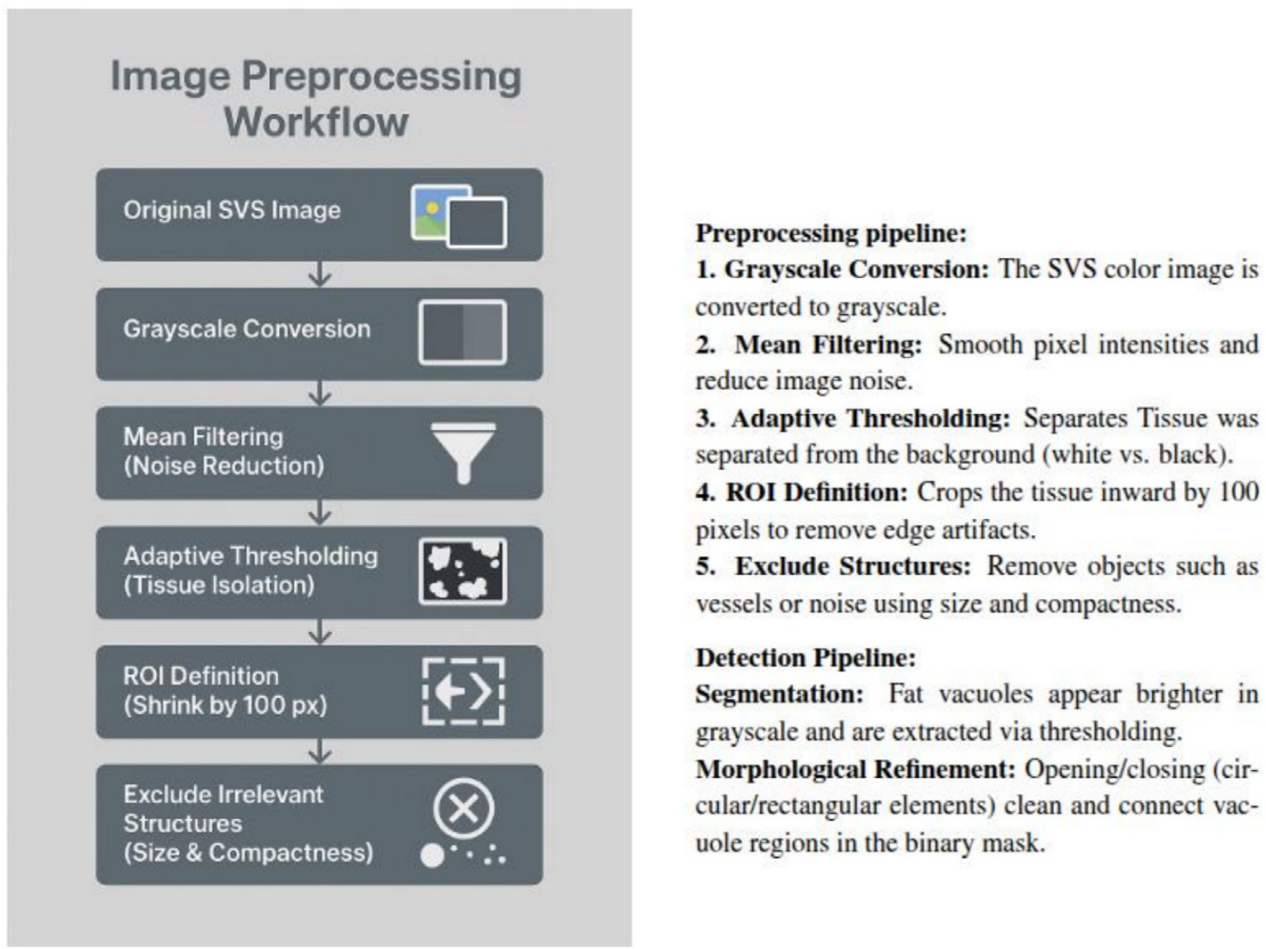
Image preprocessing and steatosis quantification workflow. The input SVS color image is converted to grayscale and smoothed by mean filtering. Adaptive thresholding separates tissue from background, and the ROI is cropped inward by 100 pixels to exclude edge artifacts. Non-relevant structures (vessels, noise) are removed by size and compactness filtering. Fat vacuoles are then extracted via intensity thresholding and refined through morphological opening/closing operations.

HALCON exposes more than 40 shape descriptors. For the validation, we standardized on four—area, roundness, circularity, and compactness—because they mirror routine pathology cues and are easy to audit in HDevelop (see Fig. 1). When appropriate during interactive review, additional descriptors (e.g., convexity, anisometry, solidity, and eccentricity) can be temporarily enabled to refine exclusion of artifacts or recovery of true vacuoles; the workflow is not restricted to a fixed feature set. (See Fig. 2.)

#### Interactive visual review and threshold adjustment (HDevelop)

During method development and qualitative checks, the pathologist and analyst jointly reviewed slides at the HDevelop console. With the full-slide overlay visible, they adjusted shape thresholds (area, diameter, roundness, circularity, and compactness) in small increments and, when useful, enabled additional descriptors to immediately include or exclude structures and tune artifact filters. Agreement was reached when the overlay reflected expert judgement; these sessions informed the final standard parameters reported in Table 2, which were then held constant for the 129-slide validation.

### Image preprocessing and steatosis quantification

H&E and Oil Red staining were performed; the automated pipeline relies exclusively on H&E slides because they are universally available in frozen-section workflows. Each SVS WSI is processed end-to-end within HALCON in the following sequence:

1. *Tissue mask generation:* RGB data are converted to grayscale using rgb1_to_gray, followed by a 5 × 5 mean filter and Otsu adaptive thresholding to produce a binary tissue mask resilient to stain saturation differences. Small speckles (<2000 μm^2^) are removed via morphological opening to eliminate debris.
2. *Artifact suppression:* The tissue mask is refined with closing and convex hull operations to bridge narrow tears, then intersected with a low-saturation mask to eliminate coverslip glare. Large voids suggestive of folds or tears are excluded using area (>1.5 × 10^6^ μm^2^) and convexity thresholds, ensuring downstream morphology is calculated only on viable tissue.
3. *Candidate detection:* Within the artifact-free tissue mask, a brightness-adaptive threshold isolates vacuole candidates. HALCON's gradient-based dyn_threshold is applied at slide-native resolution to avoid normalization that could be destabilized by staining variability.
4. *Feature extraction:* Connected components produced by the candidate mask are characterized by HALCON's built-in shape descriptors, including area, roundness, circularity, compactness, anisometry, and convexity. Only objects satisfying the macro- and microvesicular criteria listed in Table 2 proceed to quantification; residual objects are labeled as artifacts and discarded from area summation.

The combination of stain-agnostic grayscale processing and geometry-only filtering enables consistent performance across batches without color calibration, whereas artifact suppression prevents tears, folds, or blood pools from inflating the steatosis estimate.

### Statistics

Data were analyzed using SPSS (version 27.0; IBM Inc.) and R (version 4.0). Normality of distributions was assessed using the Shapiro–Wilk test. Data are presented as mean ± standard deviation or median (interquartile range, IQR), as appropriate. Continuous variables were compared using Student's *t*-test (normal distributions) or Mann–Whitney *U* test (non-normal distributions). Categorical variables were compared using the chi-square test. Analysis of variance was performed for multi-group comparisons. Statistical significance was set at *p* < 0.05.

Agreement between artificial intelligence (AI) and pathologist assessments was evaluated using multiple complementary approaches. Given non-normal distribution of steatosis values (confirmed by Shapiro–Wilk test, *p* < 0.001 for all assessors), we calculated both parametric (Pearson correlation coefficient *r* and coefficient of determination *R*^2^) and non-parametric (Spearman rank correlation ρ) metrics with 95% confidence intervals. Inter-pathologist agreement was assessed using weighted Cohen's Kappa (κ) with quadratic weights for ordinal steatosis categories (0%, 1–5%, 6–10%, 11–15%, 16–20%, 21–30%, and >30%) and root mean square error to quantify absolute disagreement magnitude between pathologists, with interpretation according to Landis and Koch^27^: κ < 0.20 (slight), 0.21–0.40 (fair), 0.41–0.60 (moderate), 0.61–0.80 (substantial), and 0.81–1.00 (almost perfect). Visual agreement was assessed by classifying cases where AI assessment was higher than, lower than, or within the range of pathologist evaluations, calculating the percentage of cases where AI fell within ±5% of mean pathologist values, and generating limits of agreement with mean plots to identify systematic estimation patterns.

## Results

### Donor data

The donor cohort (*N* = 1105) had a mean age of 57.9 years (range 1–95) with a balanced sex distribution (52.8% male). Mean anthropometrics were 83.8 kg (range 11.5–200) and 172.5 cm (range 80–205). Causes of death were cerebrovascular events (50.3%), ischemia (35.8%), and trauma (11.3%). Around 66% underwent resuscitation (mean 30 min), and 86.7% required catecholamines, which is typical for multi-organ retrieval.^28^ HTK/Bretschneider was used for preservation in 91.9%. Serology showed low hepatitis prevalence (HBV-negative 90.8%, HCV-negative 97.4%), with CMV IgG 58.9% and near-universal EBV exposure (98.5%). Common comorbidities were hypertension (44%), arteriosclerotic disease (32%), and thromboembolic conditions (33.8%). Table 1 summarizes these characteristics.

**Table 1:** Donor characteristics. Demographic, clinical, and serological data of organ donors (*N* = 1105).

Parameter	Value/Description
Age	Average: 57.9 years; Range: 1–95 years
Weight	Average: 83.8 kg; Range: 11.5–200 kg
Height	Average: 172.5 cm; Range: 80–205 cm
Sex	52.8% male, 47.2% female
Cause of death	Cerebrovascular: 50.3%; Ischemia: 35.8%; Trauma: 11.3%; Others: 0.8%
Resuscitation	∼66% resuscitated; Average: 30 min; Maximum: 3 h
Catecholamines use	86.7% required norepinephrine
Perfusion solution	HTK/Bretschneider: 91.9%; UW: ∼5%
Hepatitis B	Negative: 90.8%; Past infection: 8.1%; Others: 1.1%
Hepatitis C	Negative: 97.4%; Positive: 2.5%
CMV	IgG positive: 58.9%; IgM positive: 1%; Negative: 39.9%
EBV	Positive: 98.5%
Comorbidities	Hypertension: 44%; Diabetes: 16.8%; Tumors: 6.2%; Arteriosclerotic: 32%; Autoimmune: 3.7%; Sepsis: 4.9%; Thromboembolic: 33.8%
Substance use	Smoking: 42.4% non-smokers; Alcohol: 48.5% non-drinkers; IV drug use: 1.3%

Catecholamine use was common (86.7%), which can affect assessments of donor organ quality in the transplant setting.

### Degree of steatosis

**Fig. 3:**
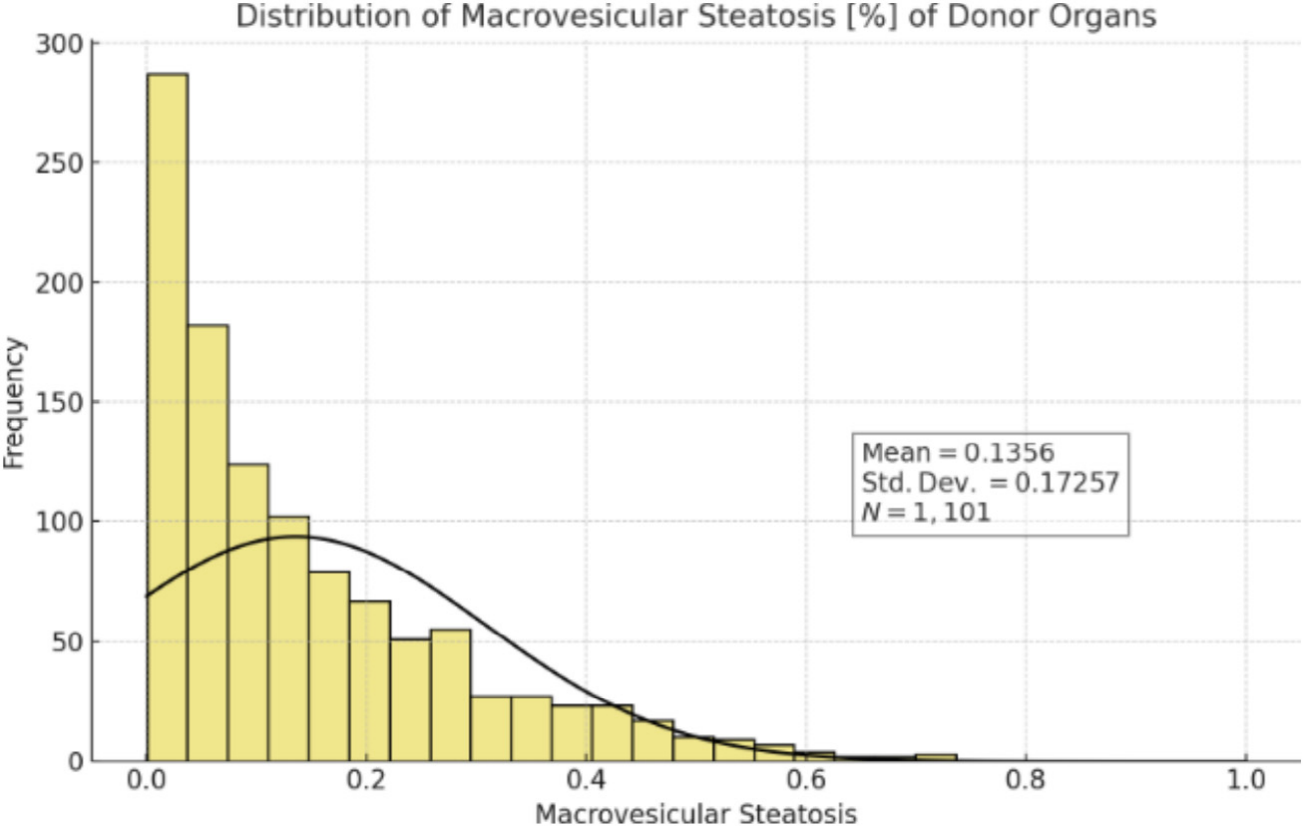
Distribution of macrovesicular steatosis in donor livers. The frequency distribution shows the percentage of macrovesicular steatosis across all donor samples (*N* = 1105). Mean steatosis was 14% with a standard deviation of 17%, with the majority of donors showing less than 20% steatosis.

Fig. 3 shows the distribution of macrovesicular steatosis. The mean was 14% (range 0–100%; SD 17%).

#### Relationship of clinical variables with macrovesicular steatosis

Clinical parameters showed significant correlations with MaS. Pearson analysis (Table 2) found positive associations with body weight (*r* = 0.194, *p* < 0.001) and BMI (*r* = 0.191, *p* < 0.001). Among labs, gamma-GT correlated most strongly (*r* = 0.201, *p* < 0.001), whereas platelets were inversely related (*r* = −0.170, *p* < 0.001).

**Table 2:** Significant correlations with macrovesicular steatosis. This table presents the significant correlations between various clinical parameters and macrovesicular steatosis (fatty liver) in donor organs. The strongest positive correlation was observed with gamma-GT (0.201), suggesting that increasing liver enzyme levels are associated with higher fat content. Weight and BMI showed moderate positive correlations (0.194 and 0.191, respectively). Hospital stay duration and platelet counts showed notable negative correlations, indicating that these values tended to decrease as liver fat increased. All correlations were statistically significant (*p* < 0.05).

Variable	Pearson correlation	Significance (2-tailed)	N
Body weight kg	0.194	<0.001	1101
Body height cm	0.082	0.006	1101
BMI $\mathrm{kg}/m^{²}$	0.191	<0.001	1101
Duration of stay before retrieval days	−0.141	<0.001	1101
Admission lab Hb g/dL	0.090	0.003	1073
Admission lab platelets /nl	−0.084	0.006	1067
Admission lab serum glucose mmol/L	0.129	<0.001	870
Admission lab gamma-GT U/L	0.201	<0.001	918
Final lab Hb g/dL	0.099	0.001	1041
Final lab leukocytes /nl	−0.089	0.004	1042
Final lab platelets /nl	−0.170	<0.001	1037
Final lab alkaline phosphatase (AP) U/L	−0.114	<0.001	888

Between-group comparisons (Table 3) were consistent: donors with high steatosis (≥30%) had higher BMI (29.84 vs. 27.60 kg/m^2^, *p* < 0.001) and gamma-GT (131.30 vs. 68.91 U/L, *p* < 0.001) than those with <30%. They stayed fewer days before retrieval (4.75 vs. 6.05, *p* < 0.001) and had lower platelets (158.60 vs. 194.10/nl, *p* < 0.001).

**Table 3:** Group statistics based on macrovesicular steatosis. This table reveals striking differences between liver donors with high and low fat accumulation. Donors with more significant fatty liver (≥30%) showed substantially higher body weight (89.49 vs. 82.78 kg), BMI (29.84 vs. 27.60), and nearly double the Gamma-GT enzyme levels (131.30 vs. 68.91 U/L). They also spent less time in the hospital before donation (4.75 vs. 6.05 days) and had notably lower platelet counts (158.60 vs. 194.10/nl). These patterns suggest metabolic differences that might influence transplant outcomes, with statistical significance across most parameters (*p* < 0.05), indicating distinct physiological profiles between these donor groups.

Variable	Macrovesicular steatosis	*N*	Mean	Std. Dev.	*p*-value
Body weight kg	≥0.30	163	89.49	18.98	<0.001
	<0.30	938	82.78	20.87	<0.001
BMI $\mathrm{kg}/m^{²}$	≥0.30	163	29.84	5.64	<0.001
	<0.30	938	27.60	5.83	<0.001
Duration of stay before retrieval days	≥0.30	163	4.75	3.39	0.00
	<0.30	938	6.05	5.42	<0.001
Admission lab gamma-GT U/L	≥0.30	133	131.30	262.35	<0.001
	<0.30	785	68.91	104.62	0.01
Admission lab INR	≥0.30	151	1.22	0.44	0.07
	<0.30	859	1.33	0.75	0.01
Final lab Hb g/dL	≥0.30	158	10.84	2.27	0.04
	<0.30	883	10.44	2.25	0.05
Final lab leukocytes/nl	≥0.30	156	12.88	5.25	<0.001
	<0.30	886	14.95	6.65	<0.001
Final lab platelets/nl	≥0.30	156	158.60	77.31	<0.001
	<0.30	881	194.10	105.31	<0.001
Final lab alkaline phosphatase (AP) U/L	≥0.30	136	84.03	55.82	0.01
	<0.30	752	101.30	76.65	0.00
Final lab INR	≥0.30	150	1.21	0.32	0.07
	<0.30	842	1.31	0.68	<0.001

Multivariable logistic regression (Table 4) identified independent predictors after adjustment. BMI (OR = 0.94, 95% CI 0.92–0.97, *p* < 0.001), gamma-GT (OR = 1.0, 95% CI 1.0–1.0, *p* < 0.001), and duration of stay (OR = 1.08, 95% CI 1.03–1.14, *p* = 0.002) remained significant. High alcohol consumption was inversely associated with steatosis in both univariate (OR = 0.39, 95% CI 0.23–0.66, *p* < 0.001) and multivariable models (OR = 0.52, 95% CI 0.29–0.69, *p* = 0.033).

**Table 4:** Univariate and multivariate analyses of variables related to macrovesicular steatosis. The analysis displays odds ratios (ORs), 95% confidence intervals (CIs), and *p*-values for both univariate and multivariate models. This comprehensive analysis identified independent predictors of steatosis, with BMI, gamma-GT, and other variables emerging as significant risk factors after adjustment for confounders.

Variable	*N*	Univariate analysis			Multivariable analysis		
		OR	95% CI	*p*-value	OR	95% CI	*p*-value
Admission lab gamma-GT U/L	1105	1.0	1.0–1.0	<0.001	1.0	1.0–1.0	<0.001
BMI $\mathrm{kg}/m^{²}$	1105	0.94	0.92–0.97	<0.001	0.94	0.92–0.97	<0.001
Final lab platelets /nl	1105	1.0	1.0–1.01	<0.001	1.0	1.0–1.01	<0.001
Alcohol “low”	1105	0.78	0.49–1.26	0.3	0.78	0.48–1.28	0.3
Alcohol “moderate”	–	0.54	0.33–0.91	0.018	0.6	0.35–1.04	0.061
Alcohol “high”	–	0.39	0.23–0.66	<0.001	0.52	0.29–0.69	0.033
Alcohol “no info”	–	0.5	0.3–0.84	0.008	0.69	0.37–1.3	0.2
Drug use “yes”	1105	1.76	0.4–5.74	0.7	1.71	0.36–6.03	0.4
Drug use “no”	–	0.75	0.16–2.57	0.7	0.95	0.20–3.51	>0.9
Duration of stay days	1105	1.07	1.02–1.2	0.70	1.08	1.03–1.14	0.002
Final urine glucose positive	1105	1.4	0.85–2.24	0.2	1.42	0.84–2.34	0.2
Final urine glucose negative	–	2.1	1.2–3.65	0.009	2.19	1.18–4.04	0.013
Admission urine glucose positive	1105	0.32	0.07–0.98	0.076	0.28	0.06–0.9	0.056
Admission urine glucose negative	–	0.25	0.06–0.7	0.022	0.26	0.06–0.75	0.031

### Whole-slide image analysis

**Fig. 4:**
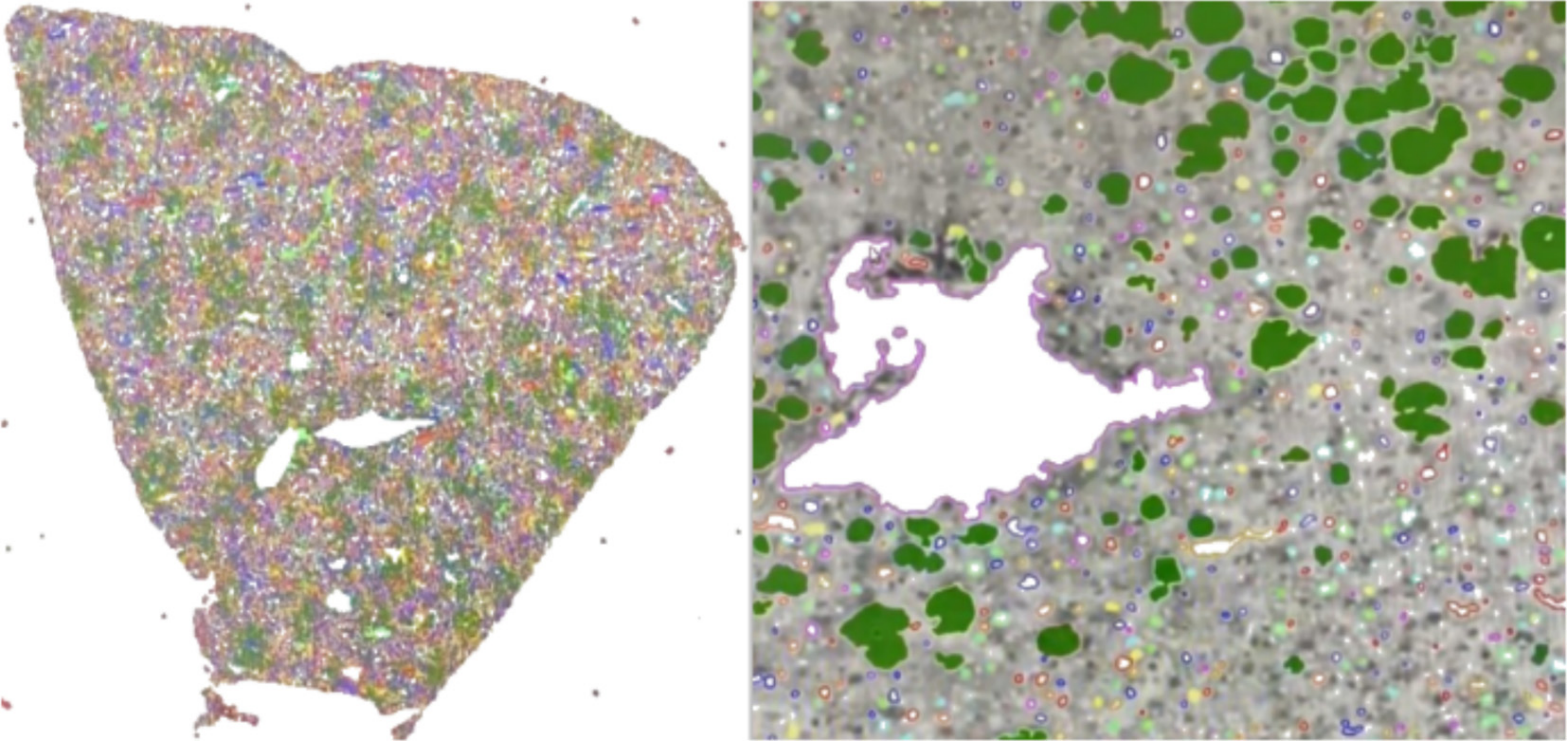
Oil Red–stained liver tissue with automated steatosis detection. Green overlays mark lipid vacuoles identified as macrovesicular steatosis, whereas vessels and staining artifacts are left unmarked. Left: WSI overview illustrates full-slide processing capacity; Right: 40× inset demonstrates pixel-level precision of vacuole segmentation, visually confirming the algorithm's ability to exclude non-fat structures. (For interpretation of the references to color in this figure legend, the reader is referred to the web version of this article.)

**Fig. 5:**
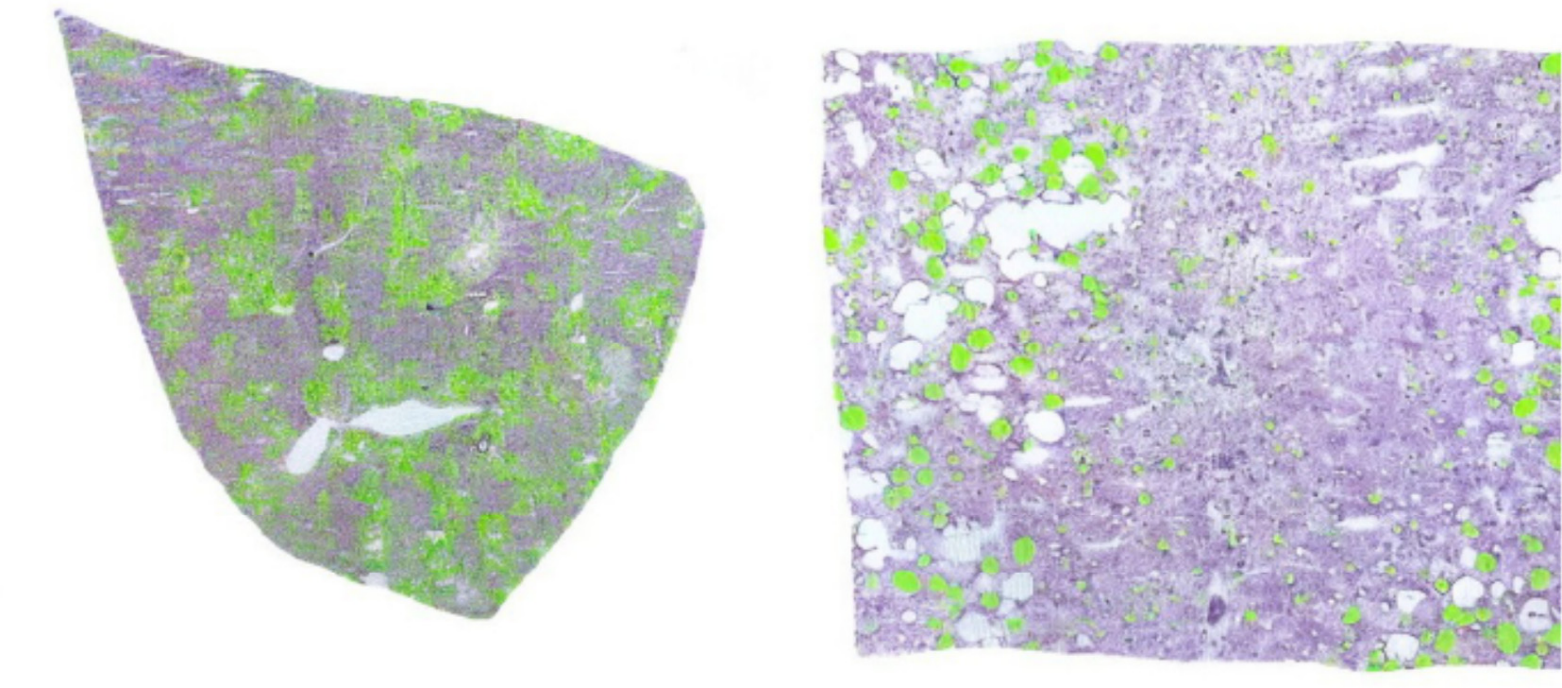
H&E-stained liver tissue illustrating feature-based pre-selection. Yellow overlays represent all round/bright structures detected in the raw thresholding step before morphological filtering. After applying area, circularity, roundness, and compactness thresholds, false-positive objects (e.g., vessels, tears) are removed (right, 20×), leaving only true fat vacuoles for quantitative analysis. (For interpretation of the references to color in this figure legend, the reader is referred to the web version of this article.)

We used HALCON to evaluate hepatosteatosis. An experienced transplant pathologist calibrated the tool for macro- and microvesicular patterns using area, circularity, roundness, and compactness. Whole-slide SVS images were analyzed in full. Fig. 4, Fig. 5 show representative outputs on different stains.

### Inter-pathologist agreement analysis

To set a baseline for human performance and context for automation, we measured inter-pathologist agreement using continuous correlations and weighted Cohen's κ on ordinal categories. This analysis used the 48 slides independently scored by all 3 liver pathologists under blinded, non-concurrent conditions.

Agreement was summarized with Pearson *r* and weighted Cohen's κ (quadratic weights) across ordinal bins (0%, 1–5%, 6–10%, 11–15%, 16–20%, 21–30%, and >30%)(Table 5).

**Table 5:** Inter-pathologist agreement analysis. Continuous (Pearson *r*) and ordinal (weighted κ) agreement metrics for 3 specialized liver pathologists on 48–49 overlapping slides. Correlations ranged from moderate (*r* = 0.582) to substantial (*r* = 0.784), with average *R*^2^ = 0.446 and weighted κ = 0.595,^27^ establishing the performance ceiling for automated methods. All correlations *p* < 0.001.

Comparison	*N*	Pearson r	95% CI	*R*^2^	RMSE (%)	Weighted κ	95% CI	Interpretation
Pathologist 1 vs. 2	49	0.582***	[0.37, 0.74]	0.339	5.9	0.499	[0.32, 0.68]	Moderate
Pathologist 1 vs. 3	48	0.619***	[0.42, 0.77]	0.383	6.4	0.557	[0.38, 0.73]	Moderate
Pathologist 2 vs. 3	48	0.784***	[0.65, 0.87]	0.615	5.7	0.729	[0.58, 0.88]	Substantial
Average	–	**0.662**	–	**0.446**	**6.0**	**0.595**	–	**Moderate**

### Inter-observer variability, human vs. machine

The application of the digital semiautomated technique to 129 WSIs resulted in a median MaS of 6.67% (IQR: 3.2–12.4%, mean: 10.1%) with values ranging from 0.18% to 39.4%. In comparison, evaluations by 3 experienced pathologists showed systematically lower median values: Pathologist 1 assessed 95 samples with a median of 5.0% (IQR: 1.0–10.0%, mean: 8.53%, range 0–40%); Pathologists 2 and 3 evaluated 49 and 48 specimens, respectively, both yielding medians of 5.0% (IQR: 2.0–8.0% and 2.0–7.5%, means: 7.16% and 6.6%, ranges 0–32% and 0–30%, respectively).

Shapiro–Wilk tests indicated non-normal distributions across all assessments (*p* < 0.001), with right-skew typical of clinical cohorts (many low-to-moderate cases and a few high-steatosis outliers). We therefore report both parametric (Pearson) and non-parametric (Spearman) correlations:

### Error analysis and failure modes

Qualitative inspection of representative AI–pathologist discrepancies, reviewed together at the HDevelop console, showed that most disagreements were resolved by interactively adjusting shape sliders (area, roundness, circularity, and compactness) while viewing the full-slide overlay. When helpful, additional HALCON descriptors can be toggled on to suppress artifacts or recover true vacuoles. The pathologist includes or excludes structures by nudging thresholds and combining features until the overlay reflects expert judgement; Supplementary Fig. S5 illustrates typical before/after examples from this side-by-side tuning.

Correlation patterns remained stable: AI vs. individual pathologists yielded *r* = 0.53–0.64 (*R*^2^ = 0.28–0.41) and AI vs. consensus reached *r* = 0.80 (*R*^2^ = 0.64), matching Spearman values within 0.02–0.04. These results sit inside the inter-pathologist envelope (*r* = 0.58–0.78; Table 6), with extended statistics provided in Table 7.

**Table 6:** Correlation between AI-derived and pathologist-assigned macrovesicular steatosis. Both parametric (Pearson) and non-parametric (Spearman) correlations reported due to non-normal distributions (Shapiro–Wilk *p* < 0.001). Individual correlations ranged from *r* = 0.526 to 0.642, whereas correlation with mean pathologist assessment reached *r* = 0.800 (*R*^2^ = 0.640). Close Pearson–Spearman concordance (Δ*r*-ρ = 0.02–0.04) validates linear relationships despite distributional skewness.

Comparison	*N*	Pearson *r* (*R*^2^)	Spearman ρ	*p*-value
AI vs. Pathologist 1	95	0.526 (0.277)	0.498	<0.001
AI vs. Pathologist 2	49	0.642 (0.412)	0.615	<0.001
AI vs. Pathologist 3	48	0.592 (0.350)	0.571	<0.001
**AI vs. Mean pathologist assessment**	**48**	**0.800 (0.640)**	**0.782**	**<0.001**

**Table 7:** Descriptive statistics of diagnostic evaluation of macrovesicular steatosis. This table provides a comprehensive statistical comparison of pathologist evaluations and digital analyses, highlighting the systematic difference, where the automated system consistently detected higher steatosis percentages than human assessors while maintaining similar distribution patterns.

Measurement	AI system	Pathologist 1	Pathologist 2	Pathologist 3
*N* (samples)	129	95	49	48
Mean (%)	10.1	8.53	7.16	6.6
Median (%)	6.67	5.0	5.0	5.0
Std. Deviation	9.93	9.2	7.73	7.0
Range	39.2	40.0	32.0	30.0
Minimum (%)	0.18	0.00	0.00	0.00
Maximum (%)	39.4	40.0	32.0	30.0
Shapiro–Wilk *W*	0.84	0.81	0.81	0.84
Shapiro–Wilk *p*	<0.001	<0.001	<0.001	<0.001

## Discussion

In this study we built and validated a HALCON-based workflow that quantifies MaS on full-resolution H&E WSIs. Without patching or special stains, the system maps steatosis across the specimen and agrees closely with expert readers. Prior work in automated steatosis quantification often relies on machine- or deep-learning; whereas accurate, many pipelines depend on large annotated cohorts, GPU hardware, and proprietary tools. Only a few, such as Madabhushi and Lee,^18^ share complete code.

Our design keeps the stack simple. Native SVS ingestion avoids extra format conversions; the thresholds in Table 2 are documented and can be reproduced; HALCON^20^ provides an accessible interface; validation includes multiple independent pathologists. These steps address reproducibility concerns raised in earlier reports.^29^ Practically, direct whole-slide loading in the XL hrunxl configuration processes multi-gigabyte files without tiling artifacts; HDevelop shows immediate overlay changes as thresholds are adjusted; and the final feature set—area, roundness, circularity, and compactness—matches routine morphological cues, keeping failure modes explainable. There is a trade-off: deep-learning models may reach *R*^2^ > 0.8, whereas our CPU-only workflow achieves *R*^2^ = 0.64 with no training data and straightforward debugging. Because inter-pathologist agreement itself ranges between *R*^2^ = 0.34–0.62 and κ = 0.595, expectations above that band must account for the variability of the reference standard, not only the algorithm.

### Interpretation of correlation metrics

The observed pattern—where overall correlation between AI and mean pathologist assessment (*R*^2^ = 0.64, *r* = 0.80) substantially exceeds individual AI-pathologist correlations (*R*^2^ = 0.28–0.41, *r* = 0.53–0.64)—merits statistical clarification. This elevation reflects variance reduction through multi-rater averaging, a well-documented phenomenon in multi-rater validation studies.^29^ When multiple raters independently assess the same cases, their measurement errors are largely uncorrelated and partially cancel upon averaging: Var(mean) = Var(individual)/*n*, where *n* = 3 raters. For our three pathologists, this reduces noise to approximately 58% of the single-rater level (√(1/3) ≈ 0.58), naturally increasing correlation with any external measure. This is not a statistical artifact but rather an expected consequence of using a more stable reference standard.

This distinction clarifies two types of validation in our study: individual correlations (*r* = 0.53–0.64) represent algorithm performance against single experts in typical clinical practice, whereas overall correlation (*r* = 0.80) represents performance against consensus assessment that approaches a theoretical “true” steatosis value by averaging out individual measurement errors. Both metrics are clinically relevant: the former indicates agreement with individual diagnostic pathologists, whereas the latter demonstrates that algorithmic estimates align well with the central tendency of expert opinion.

It is also essential to clarify that *R*^2^ (coefficient of determination) quantifies the proportion of variance explained, not the regression slope. *R*^2^ = 0.64 means “AI predictions explain 64% of variability in pathologist scores,” distinct from the regression coefficient (β), which would describe the rate of change between AI and pathologist values. For simple bivariate correlation, *R*^2^ = *r*^2^, thus *R*^2^ = 0.64 corresponds to Pearson *r* = 0.80, indicating strong positive association.

### Clinical implications of inter-pathologist variability

Table 6 places inter-pathologist agreement at κ = 0.595 with individual *r* = 0.58–0.78,^27^ underscoring that even expert reviewers disagree by ≈6%. Our AI–consensus correlation of *R*^2^ = 0.64, therefore, operates near the ceiling imposed by the reference standard rather than signaling under-performance.

Whole-slide H&E analysis minimizes sampling bias and keeps clinically decisive 10–15% shifts in view, yet residual overestimation of ∼3% must be weighed against the risk of missing high-grade steatosis in the 30% “gray zone”.^3^, ^27^ Lower variability reduces both donor discard and unexpected graft failure.

Feature selection mirrors routine pathology cues (area, circularity, roundness, and compactness), making the system a transparent assistant rather than an autonomous reader; human arbitration remains valuable in slides with overlapping vacuoles or tangential cuts.

**Fig. 6:**
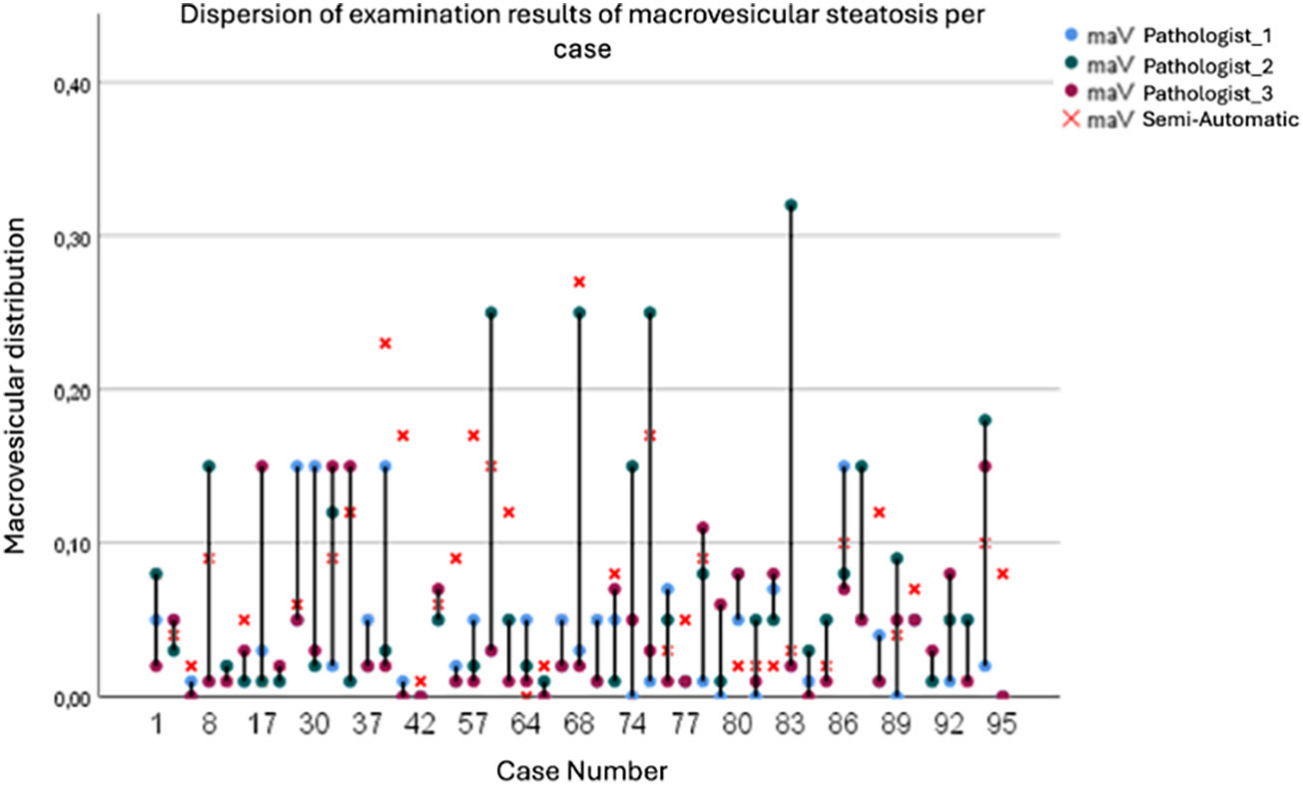

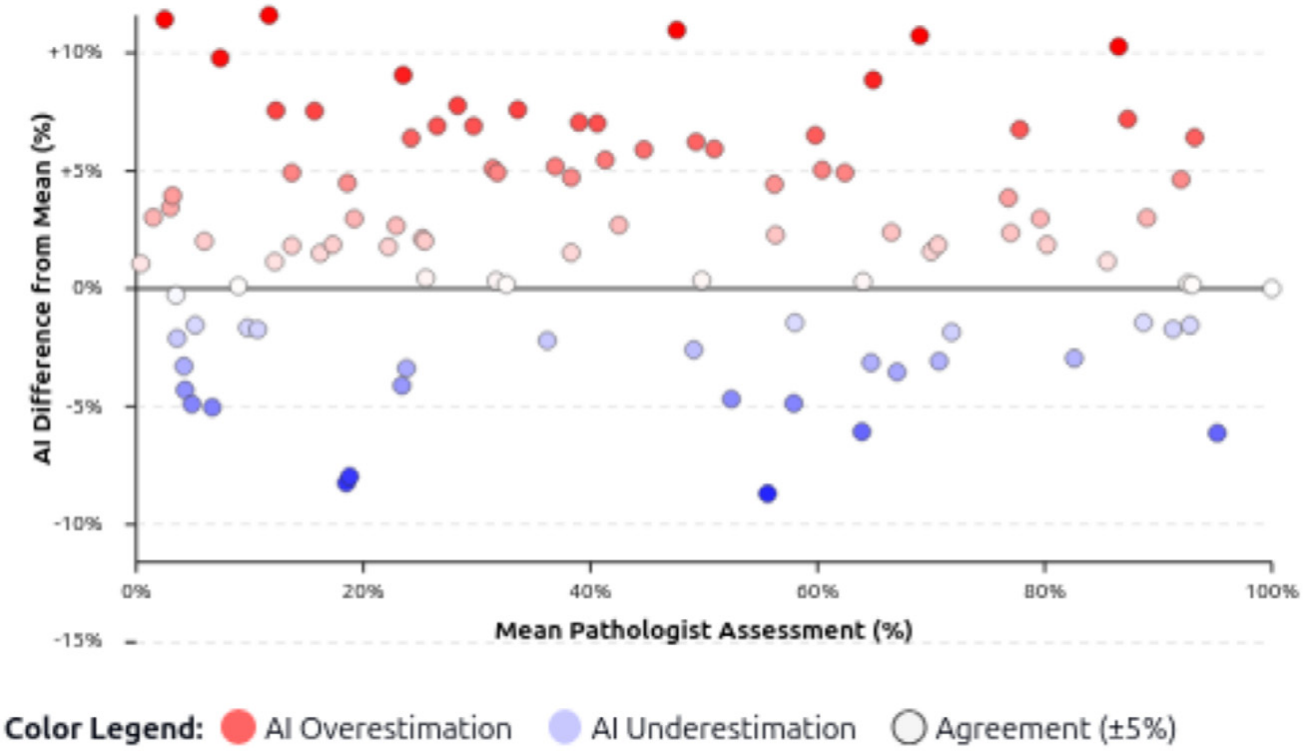
(a) Case-by-case comparison of macrovesicular steatosis. For 49 biopsies, HALCON output (red X) is shown alongside individual pathologist readings (colored dots), making intra-case variation and trends visible. Several slides showed >10% disagreement (e.g., cases 38, 39, 56, 57, 68, 88, and 95). Colors indicate different examination times. Overall, HALCON was higher in 13/49 (26.5%) and lower in 3/49 (6.1%); in the remaining 33/49 (67.3%), the HALCON value matched at least one pathologist or lay between pathologist readings. (b) Bland–Altman plot vs. Pathologist 1 (*n* = 95). The x-axis shows Pathologist 1, the y-axis the HALCON–Pathologist 1 difference. Positive values indicate HALCON overestimation; negative values underestimation. HALCON tended to overestimate at low steatosis (28% of cases), with the best agreement in the 30–60% range. In total, 66% of HALCON values fell within ±5% of Pathologist 1, indicating good—though not perfect—concordance.

The validation cohort still showed 26.5% overestimates and a 3% mean bias (Fig. 6b), highlighting the need for continual threshold calibration. Because high-grade cases were sparse (Table 3), we emphasize continuous metrics, Bland–Altman analysis, and plan multi-center expansion before reporting sensitivity/specificity at 30% MaS.

Operationally, frozen-section turnaround stays within 15 min: scanning 4–6 min, HALCON analysis median 1.15 min, review 2–4 min. Two brief training sessions covered workflow onboarding, and the CPU-only stack (≈€3800 workstation plus <€1500 academic license) keeps deployment feasible compared with GPU-dependent deep learning.

Key limitations remain: uneven pathologist coverage (95/49/48 slides), dataset-specific parameter tuning, lack of microvesicular quantification, and slide-quality variability. Broader datasets and cross-institutional validation will address these gaps while enabling further refinement of the feature-based approach.

### Future enhancements

Future development aims to broaden coverage and resilience. Detecting microvesicular steatosis will require higher-magnification analysis, tighter morphology thresholds, richer texture descriptors, and confirmation against lipid-selective stains. Additional shape cues such as eccentricity or solidity and classic texture features (e.g., Haralick descriptors^30^) could further separate vessels, artifacts, and true vacuoles, potentially aided by lightweight machine-learning classifiers. Modern deep-learning architectures, including Vision Transformers,^31^ may raise accuracy but must be deployed without sacrificing transparency. Beyond imaging alone, pairing quantitative outputs with clinical covariates could support prognostic modelling, whereas concise training materials would ease adoption in busy pathology labs. Above all, rigorous multi-center validation across scanners, staining protocols, and patient populations is essential to prove generalizability and expose edge cases that a single-center cohort cannot capture.

### Supplementary data

Supplementary Fig. S5 (files fig_s5_panel_a_overview.png, fig_s5_panel_b_intermediate.png, and fig_s5_panel_c_high_mag.png) provides multi-scale overlays illustrating how the feature-based pipeline separates macrovacuoles from portal structures and processing artifacts; the figure captions describe each panel. A supplementary glossary (supplementary_glossary.md) defines frequently used technical terms and abbreviations (WSI, adaptive thresholding, weighted κ, etc.) to support multi-disciplinary readers.

## Conclusion

Our HALCON-based workflow yields reproducible MaS quantification that correlates strongly with consensus pathologist assessments (*R*^2^ = 0.64), runs on CPUs, and fits frozen-section timelines. Using stain-agnostic morphology and interactive overlays, it serves as decision-support: it reduces—but does not remove—variability in manual grading.

Key limitations include underestimation of confluent high-grade steatosis, limited coverage of microvesicular fat, and single-center validation. Parameters may need retuning for severe cases, and multi-center studies across scanners and staining protocols are needed before routine deployment. Collaborations can also clarify how the workflow fits frozen-section meetings and which reference materials (e.g., open reference implementations, curated datasets) are most helpful for reproducible adoption.

Our findings highlight the potential of feature-based image analysis for standardizing steatosis assessments in clinical settings, yet confirm that additional evidence—particularly regarding multi-center generalizability and integration with clinical and omics biomarkers,^24^, ^32^, ^33^—is needed to refine risk stratification and support personalized graft allocation.

## Declaration of generative AI and AI-assisted technologies in the writing process

During the preparation of this work, the authors used Grammarly (https://www.grammarly.com/) in order to correct grammar, improve word choice, and find appropriate synonyms. The authors also used DeepL (https://www.deepl.com) in order to translate specific phrases and ensure accurate language conversion. Additionally, the authors used Paperpal in order to suggest precise language corrections while preserving academic context, including references, equations, technical terms, and non-English words. Finally, the authors used Writefull (https://www.writefull.com/) in order to paraphrase selected paragraphs for improved clarity and readability. After using these tools/services, the authors reviewed and edited the content as needed and take full responsibility for the content of the publication.

## Funding

Funding was provided by Dr. F. Köhler Chemie (Bensheim, Germany). They sponsored the technical equipment.

## Declaration of competing interest

The authors declare the following financial interests/personal relationships which may be considered as potential competing interests:

Prof. D.P.Hoyer reports equipment, drugs, or supplies was provided by Dr. Franz Köhler Chemie GmbH. If there are other authors, they declare that they have no known competing financial interests or personal relationships that could have appeared to influence the work reported in this paper.

## Appendix A Supplementary data

Supplementary material 1 Supplementary Figure S5 and Supplementary Glossary. Fig. S5 presents multi-scale overlays (Panel A: overview; Panel B: intermediate magnification; Panel C: high magnification) illustrating how the feature-based pipeline separates macrovacuoles from portal structures and processing artifacts. The Supplementary Glossary defines technical terms and abbreviations (WSI, SVS, H&E, HALCON, adaptive thresholding, shape descriptors, Pearson r, weighted Cohen's κ, MAE) to support multidisciplinary readers. Supplementary material 1
