# Supplementary material for: Detection of macrovesicular steatosis in hematoxylin and eosin-stained histological images of human livers: A feature-based method
Source: J Pathol Inform. 2026 Mar 27;21:100656. doi: 10.1016/j.jpi.2026.100656 (PMC13141805; doi:10.1016/j.jpi.2026.100656)
Supplement: Supplementary material 1 — Supplementary Figure S5 and Supplementary Glossary. Fig. S5 presents multi-scale overlays (Panel A: overview; Panel B: intermediate magnification; Panel C: high magnification) illustrating how the feature-based pipeline separates macrovacuoles from portal structures and processing artifacts. The Supplementary Glossary defines technical terms and abbreviations (WSI, SVS, H&E, HALCON, adaptive thresholding, shape descriptors, Pearson r, weighted Cohen's κ, MAE) to support multidisciplinary readers. [file mmc1.docx]

Supplementary Material

# Supplementary Figure S5

Supplementary Figure S5 (files `fig_s5_panel_a_overview.png`, `fig_s5_panel_b_intermediate.png`, and `fig_s5_panel_c_high_mag.png`) provides multi-scale overlays illustrating how the feature-based pipeline separates macrovacuoles from portal structures and processing artefacts; the figure captions describe each panel. A supplementary glossary (`supplementary_glossary.md`) defines frequently used technical terms and abbreviations (WSI, adaptive thresholding, weighted κ, etc.) to support multidisciplinary readers.


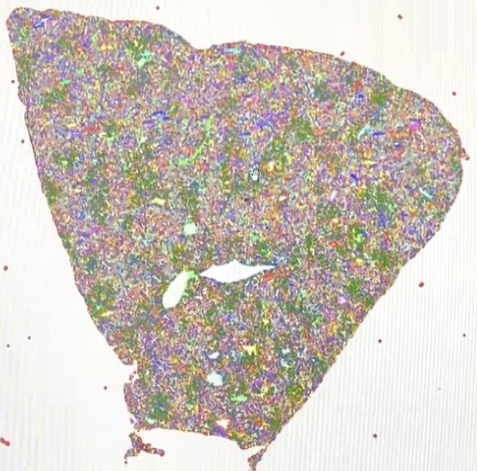


Panel A — Overview


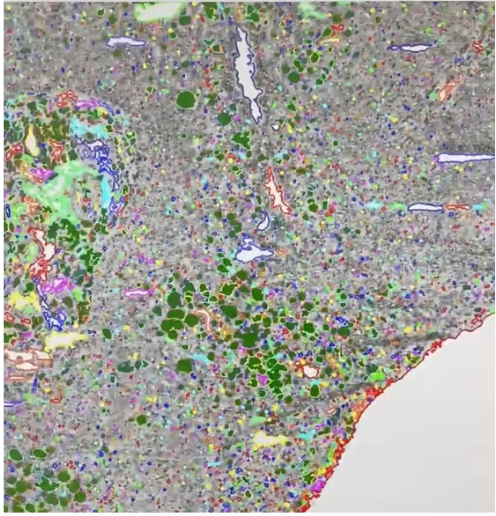


Panel B — Intermediate magnification


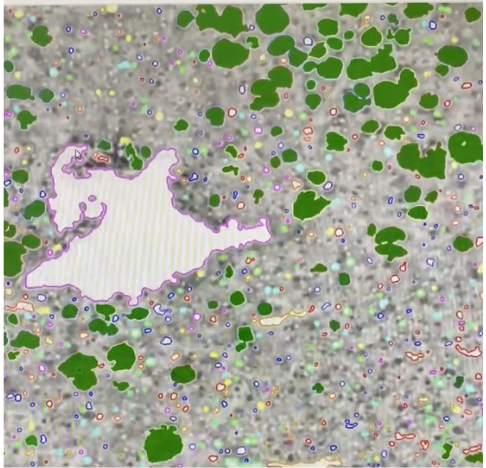


Panel C — High magnification

# Supplementary Glossary

# Supplementary Glossary

**WSI (Whole‑Slide Image):** Digitized microscopy slide at full resolution (often ≈100k × 100k px) viewable at multiple magnifications.

**SVS:** Pyramidal multi‑resolution file format used by Aperio scanners for WSIs.

**H&E:** Hematoxylin & Eosin histological stain routinely used in pathology.

**HALCON (Progress):** Commercial machine‑vision toolkit by MVTec; used here to implement feature‑based steatosis quantification.

**HDevelop / HDevEngine:** HALCON IDE and runtime; used for interactive tuning (sliders) and embedding procedures in host apps.

**`hrunxl` (XL mode):** HALCON runtime capable of opening multi‑gigabyte SVS files natively without tiling or resampling.

**Macrovesicular steatosis (MaS):** Large lipid vacuoles displacing the hepatocyte nucleus; the focus of this work.

**Microvesicular steatosis:** Numerous small vacuoles that do not displace the nucleus; not quantified by the current algorithm.

**Artifact (slide):** Non‑tissue or non‑target structures (folds, tears, glare, vessels) excluded prior to feature analysis.

**Adaptive thresholding:** Local intensity‑based thresholding (HALCON `dyn_threshold`) that adapts to regional brightness.

**Area (μm²):** Object size on the slide in square micrometres (after calibration from pixels).

**Roundness / Circularity (0–1):** Shape descriptors indicating how close an object is to a circle; help separate vacuoles from vessels.

**Compactness:** Perimeter‑to‑area‑based descriptor; higher values indicate elongated/irregular shapes (e.g., tears, vessels).

**Convexity / Solidity / Eccentricity / Anisometry:** Additional HALCON shape descriptors that can be enabled during interactive review to refine inclusion/exclusion of structures.

**Pearson r / R²:** Linear correlation coefficient and variance explained; R² = r² in the bivariate case.

**Spearman ρ:** Rank‑based (non‑parametric) correlation robust to non‑normal distributions.

**Weighted Cohen’s κ:** Agreement for ordinal ratings accounting for distance between categories (e.g., steatosis ranges).

**MAE (Mean Absolute Error):** Mean absolute difference between two percentage estimates (e.g., AI vs. pathologist).

**RMSE (Root Mean Square Error):** Square‑root of the mean of squared differences; emphasizes larger errors.

**CI (Confidence Interval):** Interval estimate expressing uncertainty around a statistic (e.g., r, κ).

**Shapiro–Wilk test:** Statistical test of normality; small p‑values indicate non‑normal distributions.

**Bland–Altman plot:** Method to visualise bias and agreement between two measurements across their range.

**Interactive visual review:** Joint session (pathologist + analyst) in HDevelop: thresholds are nudged with sliders and additional descriptors toggled on demand until the full‑slide overlay reflects expert judgement.

**CPU‑only workflow:** The pipeline runs on standard multi‑core CPUs; no GPU is required.

**ViT (Vision Transformer):** Modern deep‑learning architecture referenced for context in Discussion; not used in this study.

**Haralick features:** Classic texture descriptors (GLCM‑based) referenced as potential future enhancement.

Supplement

**Overview of Clinical Variables Collected for Organ Donors**

| Category | Variables |
| --- | --- |
| **Demographics** | Gender (m/f), Age (years), Height (cm), Body weight (kg), Body mass index (BMI, kg/m²), Blood group (AB0) |
| **Surgical Details** | *Organ removal:* abdominal, abdominal and thoracic |
| **Cause of Death** | Ischemia, cerebrovascular, tumor, trauma, other |
| **Hepatitis B Serology** | Negative, acute infection, chronic infection, vaccination protection, past infection, HBsAg carrier |
| **Hepatitis C Serology** | Positive, negative |
| **Other Serologies** | CMV: negative, acute infection, chronic infection; EBV: positive, negative |
| **ICU Stay** | Time spent in the intensive care unit (days) |
| **Laboratory Values** | Hemoglobin (Hb, g/dl), leukocytes (/nl), platelets (/nl), serum sodium (Na, mmol/L), serum glucose (mmol/L), serum creatinine (μmol/L), serum urea (mmol/L), lactate dehydrogenase (LDH, U/L), glutamate-oxaloacetate transaminase (GOT, U/L), glutamate-pyruvate transaminase (GPT, U/L), γ-glutamyl transferase (gGT, U/L), total bilirubin (μmol/L), alkaline phosphatase (AP, U/L), serum albumin (g/L), international normalized ratio (INR), C-reactive protein (CRP, nmol/L), glucose detection in urine ("positive", "negative"), Hba1c (%) |
| **Resuscitation** | Resuscitation ("yes", "no"), Resuscitation time (minutes), Catecholamines requirement ("yes", "no"), Amount of catecholamines (noradrenaline, μg/kg/min) |
| **Pre-existing Conditions** | ("yes", "no", not specified): arterial hypertension, diabetes mellitus, malignant tumor disease, arteriosclerotic disease, autoimmune disease, sepsis, thromboembolic disease, other conditions |
| **Drug Abuse** | *Alcohol, smoking, intravenous drug use:* little, moderate, a lot, no, not specified |

**Table 1: Overview of the Clinical Donor Data.** The table summarizes the 66 variables collected for each donor, categorized by the type of information.
Cases containing liver tissue from 2010 to 2019, submitted by the German Organization for Acquisitions (DSO), were identified from the Institute of Pathology's digital database. We excluded cases lacking liver tissue beyond the scope of steatosis diagnosis (e.g., cysts, space-occupying lesions, bile duct changes) without steatosis information or predating 2010 digital documentation. From the remaining 1,105 cases, a diagnostic pathologist assigned degrees of macrovesicular and microvesicular steatosis.

**Image Analysis Parameters for Automated Steatosis Detection**

| Parameter | Value (μm²) | Description |
| --- | --- | --- |
| **Image Resolution** | 0.249 | Resolution of the original WSI in micrometers per pixel |
| **MinAreaMa** | 1100 | Minimum area for macrovesicular vacuole classification |
| **MaxAreaMa** | 90000 | Maximum area for macrovesicular vacuole classification |
| **MinAreaMi** | 0.5 | Minimum area for microvesicular vacuole classification |
| **MaxAreaMi** | 1040 | Maximum area for microvesicular vacuole classification |
| **MinAreaArte** | 500 | Minimum area for artifact classification |
| **MaxAreaArte** | 9000000 | Maximum area for artifact classification |
| **MinCompactness_Arte** | 5.7 | Minimum compactness for artifacts |
| **MinCircularity** | 0.33 | Minimum circularity for fat vacuoles |
| **MinRoundness** | 0.55 | Minimum roundness for fat vacuoles |
| **MaxCompactness** | 5.5 | Maximum compactness for fat vacuoles |
| **MinDiameter (optional)** | 120 | Optional minimum diameter |
| **MaxDiameter (optional)** | 600 | Optional maximum diameter |

**Table 2: Defined Image Analysis Parameters for the Classification of Macro- and Microvesicular Steatosis.** These parameters were used with the Halcon image processing software for the semi-automated detection and quantification of steatosis in 129 histological slides of liver biopsies.
