# Supplementary material for: Detection of macrovesicular steatosis in hematoxylin and eosin-stained histological images of human livers: A feature-based method
Source: J Pathol Inform. 2026 Mar 27;21:100656. doi: 10.1016/j.jpi.2026.100656 (PMC13141805; doi:10.1016/j.jpi.2026.100656)
Supplement: Supplementary material 2 [file mmc2.docx]

# Supplement

## Table S1: Overview of the Clinical Donor Data

The table summarizes the 66 variables collected for each donor, categorized by the type of information.

| **Category** | **Variables** |
| --- | --- |
| Demographics | Gender (m/f), Age (years), Height (cm), Body weight (kg), Body mass index (BMI, kg/m²), Blood group (AB0) |
| Surgical Details | Organ removal: abdominal, abdominal and thoracic |
| Cause of Death | Ischemia, cerebrovascular, tumor, trauma, other |
| Hepatitis B Serology | Negative, acute infection, chronic infection, vaccination protection, past infection, HBsAg carrier |
| Hepatitis C Serology | Positive, negative |
| Other Serologies | CMV: negative, acute infection, chronic infection; EBV: positive, negative |
| ICU Stay | Time spent in the intensive care unit (days) |
| Laboratory Values | Hemoglobin (Hb, g/dl), leukocytes (/nl), platelets (/nl), serum sodium (Na, mmol/L), serum glucose (mmol/L), serum creatinine (μmol/L), serum urea (mmol/L), lactate dehydrogenase (LDH, U/L), glutamate-oxaloacetate transaminase (GOT, U/L), glutamate-pyruvate transaminase (GPT, U/L), γ-glutamyl transferase (gGT, U/L), total bilirubin (μmol/L), alkaline phosphatase (AP, U/L), serum albumin (g/L), international normalized ratio (INR), C-reactive protein (CRP, nmol/L), glucose detection in urine ("positive", "negative"), Hba1c (%) |
| Resuscitation | Resuscitation ("yes", "no"), Resuscitation time (minutes), Catecholamines requirement ("yes", "no"), Amount of catecholamines (noradrenaline, μg/kg/min) |
| Pre-existing Conditions | ("yes", "no", not specified): arterial hypertension, diabetes mellitus, malignant tumor disease, arteriosclerotic disease, autoimmune disease, sepsis, thromboembolic disease, other conditions |
| Drug Abuse | Alcohol, smoking, intravenous drug use: little, moderate, a lot, no, not specified |

## Table S2: Defined Image Analysis Parameters for the Classification of Macro- and Microvesicular Steatosis

| **Parameter** | **Value (μm²)** | **Description** |
| --- | --- | --- |
| Image Resolution | 0.249 | Resolution of the original WSI in micrometers per pixel |
| MinAreaMa | 1100 | Minimum area for macrovesicular vacuole classification |
| MaxAreaMa | 90000 | Maximum area for macrovesicular vacuole classification |
| MinAreaMi | 5 | Minimum area for microvesicular vacuole classification |
| MaxAreaMi | 1040 | Maximum area for microvesicular vacuole classification |
| MinAreaArte | 500 | Minimum area for artifact classification |
| MaxAreaArte | 9000000 | Maximum area for artifact classification |
| MinCompactness_Arte | 5.7 | Minimum compactness for artifacts |
| MinCircularity | 0.33 | Minimum circularity for fat vacuoles |
| MinRoundness | 0.55 | Minimum roundness for fat vacuoles |
| MaxCompactness | 5.5 | Maximum compactness for fat vacuoles |
| MinDiameter (optional) | 120 | Optional minimum diameter |
| MaxDiameter (optional) | 600 | Optional maximum diameter |

**Table S2: Defined Image Analysis Parameters for the Classification of Macro- and Microvesicular Steatosis.** These parameters were used with the Halcon image processing software for the semi-automated detection and quantification of steatosis in 129 histological slides of liver biopsies.
